# Psychometric properties of the Chinese version of the empathy quotient among Chinese minority college students

**DOI:** 10.1186/s12991-018-0209-z

**Published:** 2018-09-19

**Authors:** Yanjun Zhang, JiuYu Xiang, Jianlin Wen, Wei Bian, Liangbin Sun, Ziran Bai

**Affiliations:** 1 College of Teacher Education, Zhejiang Normal University, Jinhua, zhejiang Province, People’s Republic of China; 2 School of Marxism, Wuhan University, Wuhan, Hubei Province, People’s Republic of China; 3 School of Marxism, Chongqing University, No. 174 Shazhengjie, Shapingba, Chongqing, 400044 People’s Republic of China; 4 Southwest Eye Hospital, Third Military Medical University, Chongqing, People’s Republic of China; 5 School of Journalism and Communication, Chongqing University, Chongqing, People’s Republic of China

**Keywords:** Empathy, Empathy quotient, Reliability, Validity, Chinese minority nationality college students

## Abstract

**Background:**

When the minority college students from the ethnic minority communities come to study in Chinese Han region, they encounter adapting difficulties of culture and socio-psychology, in which empathy plays a crucial role. Current instruments used to measure empathy have many limited effectiveness. The empathy quotient (EQ) scale which has been validated in many countries was explicitly designed for clinical applications and was intended to be sensitive to a lack of empathy. This study is to develop a complete Chinese version of the EQ scale and to assess its reliability and validity among Chinese minority college students in the Han Chinese region.

**Methods:**

A total of 1638 Chinese minority college students in the Han region were selected and were randomly divided into two groups. One group of 818 students took part in the implementation of the exploratory factor analysis while the other group of 820 students participated in the confirmatory factor analysis.

**Results:**

Twenty-nine items of the EQ were retained based on the factor analysis and four factors were extracted: self-awareness, cognitive empathy, social skills, and emotional reactivity, which can explain 51.793% of the total variance. The factors of the EQ scale were significantly correlated with each other, with the correlation coefficient ranging from 0.316 to 0.563. The coefficient of internal consistency (Cronbach’s *α*) was 0.824 for the total scale and ranged from 0.640 to 0.818 for the subscales. Confirmatory factor analysis proved that the measured data fitted well with the hypothesized four-factor model. All of the items in the scale fitted the model well, and the point-measure correlation coefficient had acceptable consistency.

**Conclusions:**

The refined 29-item Chinese version of the EQ possesses good reliability and validity, and can be applied in assessing empathy among Chinese minority college students.

## Introduction

As an important ability for social communication, empathy in the broadest sense refers to the reactions of one individual to the observed experiences of another [1]. For its clinical implication, empathy helps to accurately represent others’ psychological states and, therefore, enables self-control and adequate behavior in social contexts [2]. Researchers pointed out that the impairment of empathy may cause some mental psychiatric conditions including antisocial personality disorders and psychopathy [3, 4]. When the minority college students come to study in the Chinese Han region, they will inevitably find themselves immersed in a brand-new environment in which their interpersonal relationships and efforts to assimilate with the students of the Han nationality require an adapting process of psychology. For these minority college students, these 4 years of college life can also be viewed as a form of immigration. Language obstacles and a diverse culture make it difficult for them to understand what are taught in the class. Some of the minority students can successfully assimilate themselves into the new groups, but quite a number of them exhibit an abortive adaptation. During their adaptation, stressors like having trouble in understanding what teachers say, fearing about passing examinations, and the feeling of being excluded from groups will finally lead to psychological disorders such as anxiety, depression, and autism. In this study, we chose Uyghur and Hui nationalities as our study samples because Uyghur and Hui nationalities are typically representative with big population and widely distributed people among China’s 55 ethnic minorities. Accounting for a large proportion of the minority college students in China, the two nationalities share the Islamic faith and their cultures are noticeably different from Han culture.

In the multicultural adaptation of Chinese minority college students, empathy helps to abate the cultural anxiety that emerges from the course of interpersonal communication because it could be viewed as the “glue” of the social world, drawing us to help others and stopping us from hurting others [5].

Although empathy without question plays a crucial role in interpersonal relationships, it is difficult for researchers to agree on a consistent definition and use of the term empathy [6]. Traditionally, researchers in this area have fallen into two camps: those who conceptualized empathy as more cognitive and those who conceptualized it as more affective [7]. However, a consensus has recently been reached in that both approaches have been essential to conceptualizing empathy and recognizing its multidimensional nature: the cognitive and affective approaches cannot be easily separated. Due to the historical divergence of recognizing the nature of empathy, instruments of various kinds have been developed to measure empathy. Among them, self-report questionnaires are one of the most widely used instruments because they are easy to use and can access multiple dimensions more straightforwardly than can other methods [8]. Some questionnaires for measuring empathy were developed, but it is doubtful that many of them are suitable instruments for measuring empathy. Here, we illustrate three typical types of questionnaires for measuring empathy: the empathy scale [9], the Questionnaire Measure of Emotional Empathy (QMEE) [10], and the Interpersonal Reactivity Index (IRI) [11].

The empathy scale was intended to measure empathy in a cognitive sense, but it later was found to have four independent factors: social self-confidence, even-temperedness, sensitivity, and nonconformity [12]. Of these four factors, only sensitivity is thought to be directly relevant to empathy; so, the empathy scale was not considered a pure measure of empathy but sort of a measure of social skills [13]. The Questionnaire Measure of Emotional Empathy (QMEE) was designed to assess an individual’s tendency to react strongly to another’s experience [10]. The authors of the QMEE suggest that the split-half reliability is high (0.84), which indicates the items are likely to tap a single construct, but this single construct may be emotional arousability to the environment in general, rather than to people’s emotions in particular [14]. The IRI comprises four subscales: perspective-taking, empathic concern, personal distress, and fantasy. Because three of the four factors are directly relevant to empathy, the IRI was once thought to be the best way to measure empathy. But items of the fantasy subscale that state, “I daydream and fantasize, with some regularity, about things that might happen to me” and items of the personal distress subscale saying, “In emergency situations, I feel apprehensive and ill at ease” indicate that the IRI may measure processes broader than empathy and that these factors are not empathy itself [5].

To address the deficiencies of the existing questionnaires, Baron-Cohen and Wheelwright [5] developed a new self-report measure of empathy: the empathy quotient (EQ). The EQ was explicitly designed for clinical applications and was intended to be sensitive to a lack of empathy as a feature of psychopathology. The original, the Japanese [15], the French [2], the Korean [8], the Italian [16], and the Chinese [17] versions of the EQ have been validated in samples of university students and of the general population, in adults with high-functioning autism or Asperger’s disorder, and with depersonalization disorder [18]. The aim of our study was to develop a Chinese version of the EQ and to establish its psychometric properties based on Chinese minority college students, a potentially useful assessment in working with Chinese minority college students who may suffer from mental disorders during the process of this typical immigrant adaptation.

## Methods

### Objects

A convenience sampling of 1650 Uyghur and Hui nationality college students (freshmen to seniors) from two Chinese universities (Chongqing University and Zhejiang Normal University) were recruited in May 2016. Approval for this study was obtained from the office of social science of the two universities. Inclusion criteria were as follows: (1) from Xinjiang Autonomous Region; (2) aged 17 years or older; (3) can read and understand Mandarin; (4) were not taking any anti-anxiety or antidepressant medication; and (5) did not have any other systematic diseases. The medical records of the students had been collected from the students file.

All questionnaires were returned, and there were no students refusing to participate. But twelve were incorrectly completed, leaving a total sample of 1638 subjects. 818 participants were randomly selected for the implementation of the exploratory factor analysis (EFA). The remaining 820 participants were arranged to participate in the confirmatory factor analysis (CFA).

A demographic data sheet including the age, gender, grade, and geographic area was also collected in the beginning of the study.

### Instruments

#### Empathy quotient (EQ)

The empathy quotient (EQ), prepared by Professor Baron-Cohen and Professor Wheelwright [5] in 2004, is a scale specifically used to test the status of empathy among adults. It was organized into the three subscales of cognitive empathy, emotional reactivity, and social skill subscales. The original scale consists of 60 items, including 40 scoring items and 20 filler items. All the items were measured on a 4-point Likert-type scale ranging from complete agreement and half agreement to half disagreement and total disagreement. The final score was the total of all scoring items. The highest score was 80 (best EQ), while the lowest was 0 (worst EQ).

### Translation and adaptation

We developed the Chinese version of the empathy quotient scale after obtaining the permission from Professor Baron-Cohen and his team, followed by a standard forward and backward translation procedure [19]. Firstly, two professional translators were employed to translate the EQ into Chinese, and panelists (including two psychologists and three education experts) were invited to conduct language and culture adjustments, perform content evaluation of the preliminary scale, and determine the first draft of the scale. The back-translation was conducted by two bilingual experts to translate the first draft into English, make comparisons with the original scale, find the differences, make corresponding amendments to the translated first draft, and ultimately reach a consistent opinion. The whole process was conducted rigorously to ensure semantic, idiomatic, experiential and conceptual equivalence to respect cultural considerations.

Fifty Uyghur college students were then selected to participate in the pretest, after which further amendments were made according to the results, and the final Chinese (language) version of the EQ was developed.

### Data collection

The investigators directly distributed the scale to the participants of study, informed them of the purpose and process of this research, and had them sign the informed consent. Next, the objects of study carefully filled out the form item by item. At the time of collecting questionnaire, investigators immediately checked whether the questionnaire was entirely filled in. In case of any missing items, it was required to have them refilled at once and the questionnaire was collected only after proper checks and verifications were finalized. One week after the first round of investigation, 50 participants were randomly selected from the 818 participants to conduct the second round of filling out the questionnaire, for the purpose of testing the test–retest reliability of the questionnaire.

### Data analysis

Data analysis was carried out by the SPSS 17.0 software package and checked by two researchers to ensure consistency. AMOS 21.0 was applied to test the confirmatory factor analysis. Descriptive statistics were used to summarize sample characteristics and the Kolmogorov–Smirnov test was used to examine the normal distribution of the data. Construct validity was statistically tested by means of principal component factor analysis with varimax rotation.

The reliability analysis of the EQ was tested by calculating the Cronbach’s *α* and test–retest reliability by intra-class correlation coefficient. A Cronbach’s *α* ≥ 0.70 was considered adequate [20].

Construct validity was evaluated by factor analysis. Regarding factor analysis, the principal components method was used to extract common factors based on the eigenvalues > 1 criterion and also scree plots, and the varimax rotation method to reveal relations (factor loadings) between common factors and items [21].

A content validity index (CVI) was used to describe the content validity. The expert panel was asked to score each item regarding the relevance to the total questionnaire on a 4-point scale of 4 = very relevant, 3 = quite relevant, 2 = somewhat relevant, and 1 = not relevant. The CVI was calculated by the percentage of items receiving a rating of 3 or 4, and a CVI value exceeding 0.80 indicated good content validity [20].

Convergent validity was evaluated by the correlation coefficient between the scores of every subscale and the total score.

A confirmatory factor analysis was conducted to examine the EQ structure to see if the factor structure reflected the proposed theoretical model. Statistical methods were used to test the fit of the model: *χ*^2^/df, the goodness of fit index (GFI), adjusted goodness of fit index (AGFI), incremental fit index (IFI) value, comparative fit index (CFI), and Tucker–Lewis Index (TLI), and root mean square error of approximation (RMSEA). A *χ*^2^ test with *P* > 0.05 shows a good model fit. Also, A model with 1 < *χ*^2^/df < 5, IFI > 0.9, GFI > 0.9, AGFI > 0.9, CFI > 0.9, TLI > 0.9, RMSEA < 0.05 suggested a good model fit. Additionally, average variance extracted (AVE) was calculated from model estimates using the AVE formula given by, and the AVE for all exceeded the recommended level of 0.50. The maximum shared squared variance (MSV), and average shared squared variance (ASV) was less than AVE.

## Results

### Demographic data of the participants

Of all the 1638 participants who returned valid questionnaires, 941 students are with Uyghur nationality, 697 students are with Hui nationality. 884 (54.0%) of the participants are males, with an age range of 17–24, and 936 (57.1%) are from urban areas; 909 (55.5%) of the participants are only child.

A total of 818 participants were randomly selected for the implementation of exploratory factor analysis (EFA) while the remaining 820 participants participated in confirmatory factor analysis (CFA). Among the EFA group, there were 431 males and 387 females, with an average age of (20.69 ± 2.12). Among the CFA group, 453 were males and 367 were females, with an average age of (21.73 ± 3.24). There was no statistical significance between the two groups with regard to the demographic data.

### Construct validity

**Fig. 1:**
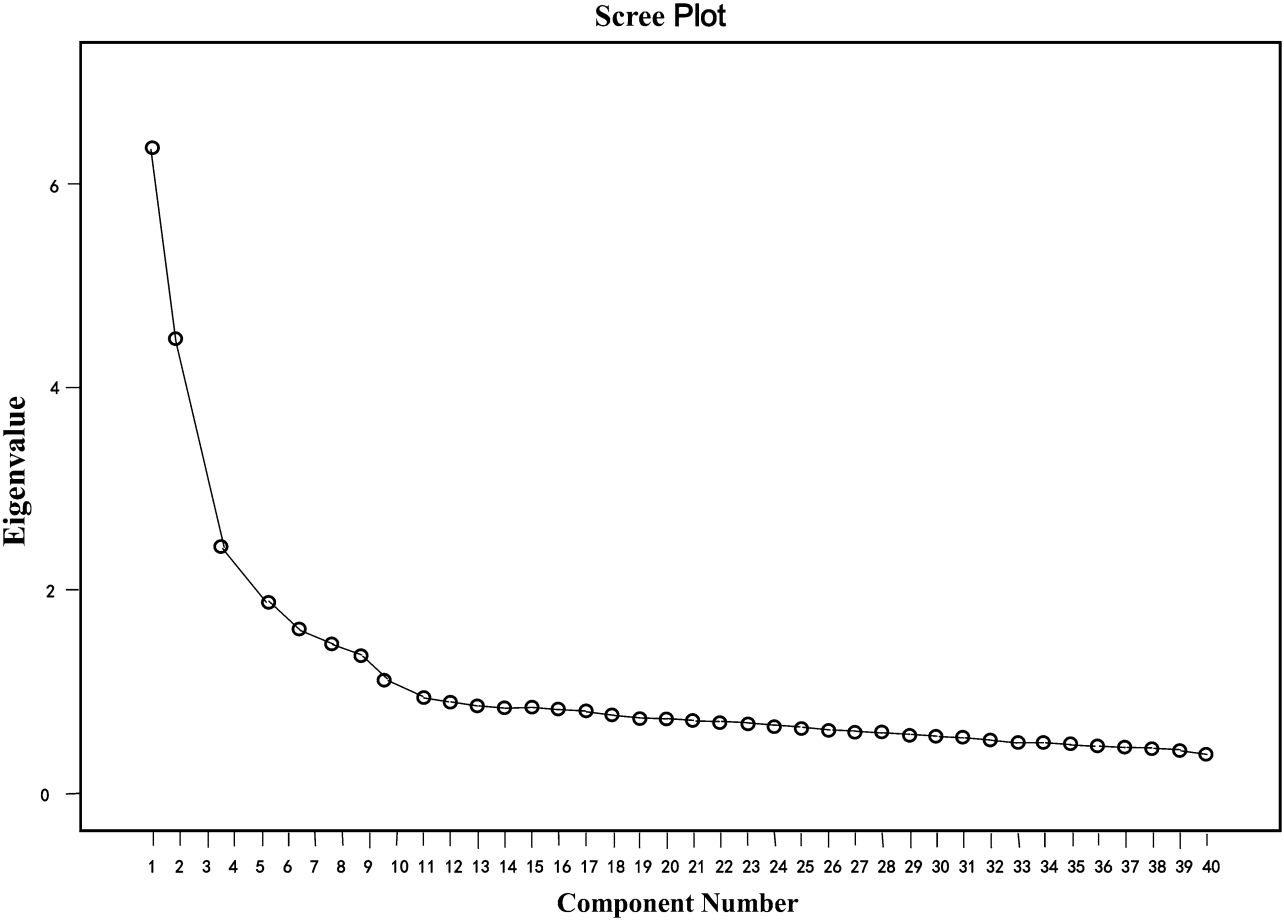
A scree plot illustrating the factor loadings of the EQ questionnaire

The Kaiser–Meyer–Olkin (KMO) score for the Chinese version of the EQ scale was 0.888 and the Bartlett’s test for sphericity was significant (*P* < 0.001), suggesting that the EQ scale was suitable for principal component analysis (PCA). As for the factor extraction, the factors with their extraction characteristic values of PCA greater than 1 are selected. Regarding the factor rotation method, the method combining both orthogonal rotation and oblique rotation is adopted. During the process of exploratory factor analysis, by grounding the factor analysis on the entry deletion criteria, those entries with factor loading smaller than 0.4, multiplicity factor loading, and unexplained dimensionality of belonging were deleted or retained after the panel discussion. Moreover, the factor analysis was conducted a second time for each deleted entry. As a result, four factors were extracted and 29 items ultimately retained. The scree plot suggested generating a four-factor model (Fig. 1).

The first factor, “cognitive empathy,” which refers to the process of investigation in terms of recognizing and understanding the emotional feelings of others, accounted for 18.994% of the total variance. The second factor, “self-consciousness,” referring to the process of investigation in terms of understanding the self-competence from individuals, accounted for 15.260% of the total variance. The third factor, “emotional empathy,” the common experience of the emotional feelings of others, accounted for 13.386% of the total variance. Finally, the fourth factor, “social skills,” or the skills and abilities manifested during the interactions between individuals and others, accounted for 13.073% of the total variance. See Table 1.

**Table 1:** Factor loading, eigenvalues, and percent of variance for EQ scale items emerging from the principal components analysis (*n* = 818)

Items	Factors			
	Cognitive empathy	Self-consciousness	Emotional empathy	Social skills
1. EQ36				
Good at understanding others	0.787			
2. EQ26				
Quick to feel others are uncomfort	0.760			
3. EQ41				
Sensitive to others’ feelings	0.756			
4. EQ19				
Insightful to others’ talk	0.743			
5. EQ54				
Sensitive to others’ talk intention	0.693			
6. EQ52				
Tune into how someone feels	0.588			
7. EQ58				
Good at prediction	0.579			
8. EQ55				
Sensitive to others’ talk intention	0.568			
9. EQ44				
I can sense if I am intruding	0.567			
10. EQ01				
Sensitive to others’ intention	0.536			
11. EQ15				
Focus on my own thoughts in talk		0.711		
12. EQ34				
Regard my bluntness as rudeness		0.682		
13. EQ27				
Say offendence		0.668		
14. EQ28				
Reply someone truthfully		0.676		
15. EQ30				
Being often told unpredictable		0.668		
16. EQ31				
Enjoy being the center		0.642		
17. EQ24				
Like impulsion		0.555		
18. EQ29				
Can’t always see offendence cause		0.527		
19. EQ06				
Enjoy caring for other people			0.779	
20. EQ50				
Emotionally detached with a film			0.727	
21. EQ38				
Feel upsets to see animals in pain			0.683	
22. EQ42				
Get upset if see sufferings			0.632	
23. EQ21				
Hard to find upset			0.597	
24. EQ59				
Involved with a friend’s problems			0.519	
25. EQ08				
Hard to know what to do				0.750
26. EQ35				
Don’t tend to find confusion				0.641
27. EQ04				
Difficult to explain to others				0.627
28. EQ48				
People say I am insensitive				0.583
29. EQ33				
Enjoy discussing about politics				0.548
Eigenvalues	4.679	2.213	2.159	1.857
Variance explained	18.994%	15.260%	13.386%	13.073%

Only factor loading values over 0.4 was listed here

### Reliability

The result showed that Cronbach’s *α* of the EQ total scale was 0.824, and Cronbach’s *α* of every subscale ranged between 0.714 and 0.818. The test–retest reliability of total scale was 0.896, and the test–retest reliabilities of every subscale ranged between 0.718 and 0.943 (see Table 2).

**Table 2:** Correlation between scores for each EQ scale and the total EQ scale score

	Self-consciousness	Cognitive empathy	Social skills	Emotional empathy
Self-consciousness	1			
Cognitive empathy	0.316^a^	1		
Social skills	0.563^a^	0.383^a^	1	
Emotional empathy	0.494^a^	0.474^a^	0.375^a^	1
Total	0.827^a^	0.544^a^	0.646^a^	0.525^a^

^a^*P *< 0.01

### Content validity

The expert panel was invited to review the contents of the scale, and to make language and culture adjustments to entries so as to make them relevant to the expression of Chinese people. All the experts agreed that the Chinese version of the EQ scale was suitable for the determination of empathy status among Uyghur college students and the representativeness of entries was fine. And the CVI was 0.928, indicating adequate content validity.

### Convergent validity

The correlation coefficient between each subscale of the EQ was significant, ranging between 0.316 and 0.563 and indicating moderate correlation. The correlation coefficient between each subscale and the total score ranged between 0.525 and 0.827, indicating high correlation (see Table 2).

### Confirmatory factor analysis (CFA)

**Fig. 2:**
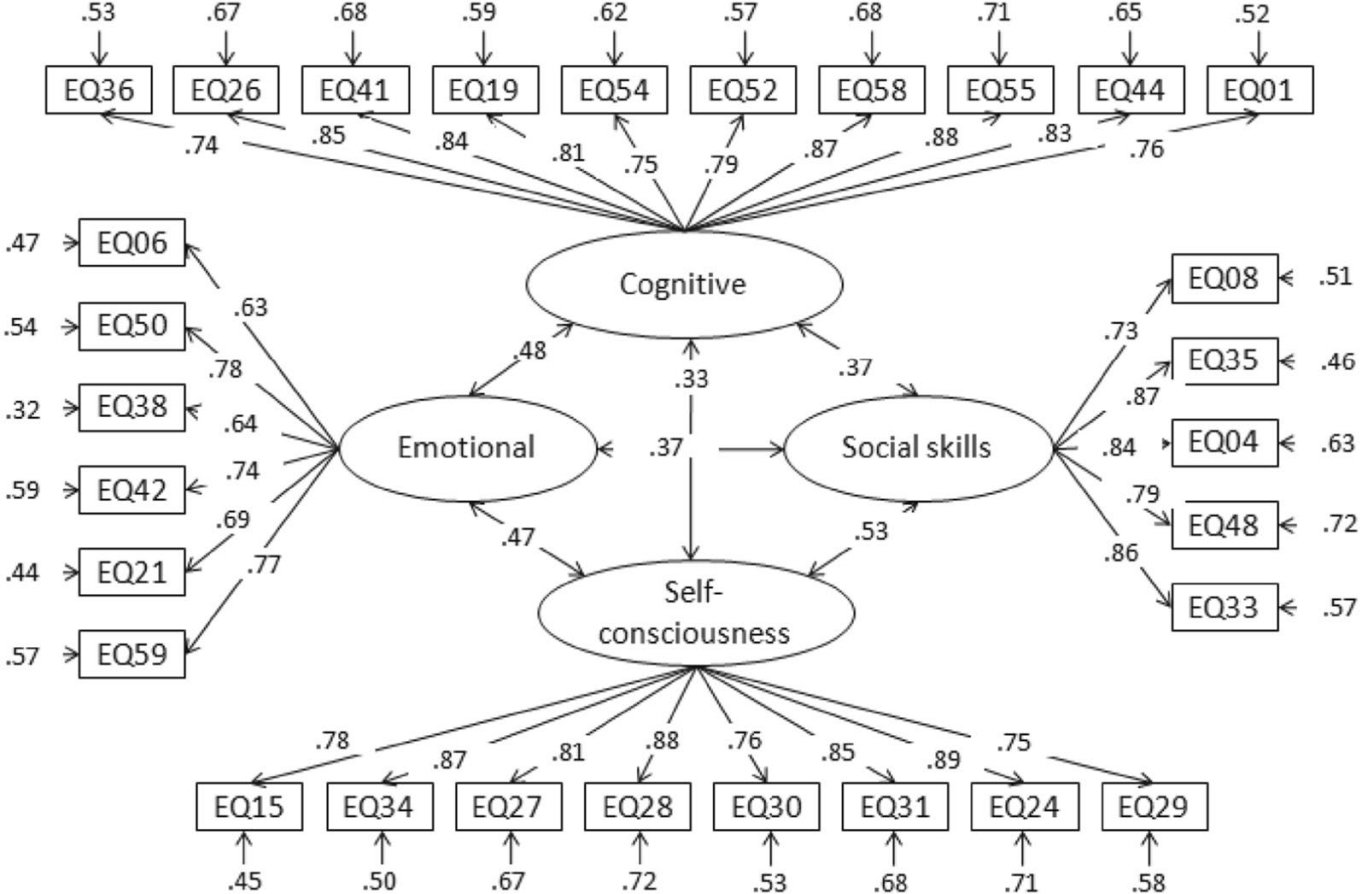
Four-factor model of EQ questionnaire with standardised parameter estimates and factor intercorrelations

As indicated in the result, *χ*^2^/df = 2.51 < 5, and the goodness of fit index (GFI), adjusted goodness of fit index (AGFI), incremental fit index (IFI) value, comparative fit index (CFI), and Tucker–Lewis Index (TLI) were 0.942, 0.928, 0.920, 0.919, 0.909, respectively. All of them were greater than 0.9, and the root mean square error of approximation (RMSEA) was 0.043 < 0.06. The results showed that the EQ scale fitted well into a four-factor model and all items were found to contribute significantly to their respective latent constructs. The four-factor model path diagram with standardized parameter estimates and factor inter-correlations is shown in Fig. 2.

The calculation of AVE and MSV showed that the AVE for all exceeded the recommended level of 0.50 and the MSV was less than AVE (shown in Table 3).

**Table 3:** Results for the measurement model

Construct	Items	Factor loading	ASV	MSV	AVE	CR
Self-consciousness	EQ15	0.711	0.220	0.317	0.508	0.891
	EQ34	0.682				
	EQ27	0.668				
	EQ28	0.676				
	EQ30	0.668				
	EQ31	0.642				
	EQ24	0.555				
	EQ29	0.527				
Cognitive empathy	EQ36	0.787	0.157	0.225	0.502	0.909
	EQ26	0.760				
	EQ41	0.756				
	EQ19	0.743				
	EQ54	0.693				
	EQ52	0.588				
	EQ58	0.579				
	EQ55	0.568				
	EQ44	0.567				
	EQ01	0.536				
Emotional empathy	EQ06	0.779	0.201	0.317	0.517	0.810
	EQ50	0.727				
	EQ38	0.683				
	EQ42	0.632				
	EQ21	0.597				
	EQ59	0.519				
Social skills	EQ08	0.750	0.203	0.244	0.570	0.797
	EQ35	0.641				
	EQ04	0.627				
	EQ48	0.583				
	EQ33	0.548				

### Comparison of EQ scale scores between different genders

A *T* test with independent samples was conducted among all the scores of the participants. The result showed that the median scores for the total EQ as well as “self-consciousness,” “social skills,” and “emotional empathy” subscales were significant higher in female college students compared with male college students (*P *< 0.001; see Table 4).

**Table 4:** Score comparison of the Chinese version of the empathy quotient (EQ) scale for male and female college students ($\overline{X}$ ± s)

Subscales	Total	Male	Female	*t*	*P*
	(*n* = 1638)	(*n* = 884)	(*n* = 754)		
Self-consciousness	10.0 ± 0.4	9.1 ± 0.4	11.0 ± 0.4	− 6.24	< 0.001
Cognitive empathy	6.3 ± 0.7	6.1 ± 0.7	6.4 ± 0.7	− 1.68	0.092
Social skills	3.3 ± 0.6	2.9 ± 0.9	3.7 ± 0.5	− 5.26	< 0.001
Emotional empathy	4.3 ± 0.9	4.0 ± 0.8	4.6 ± 0.9	− 4.66	< 0.001
Total	23.9 ± 0.6	22.2 ± 0.4	25.7 ± 0.5	− 6.89	< 0.001

### Comparison of different EQ models

The original EQ (60 items) has 40 items that measure empathy as a single construct and another 20 filler items. In the new Chinese EQ, 29 entries and 4 factors were ultimately retained which include three of the previous factors F1 (10 items): cognitive empathy, F3 (6 items): emotional empathy, and F4 (5 items): social skills, and one more added factor F2 (8 items): self-consciousness. The differences between the items available for the English version and the Chinese version are as the following Table 5.

**Table 5:** Differences of EQ structural models and CFA results

EQ model	Factors (item number) Cronbach’s *α*				CFA results			
	F1	F2	F3	F4	*χ*^2^/df	CFI	TLI	RMSEA
Baron-Cohen and Wheelwright [5]	EM (40) 0.86	–	–	–	4.383	0.73	0.71	0.076
Lawrence et al. [18]	CE (11) 0.87	ER (11) 0.69	SS (6) 0.57	–	4.577	0.84	0.83	0.078
Wakabayashi et al. [33]	EM (22) 0.86	–	–	–	5.744	0.86	0.84	0.090
Muncer and Ling [34]	CE (5) 0.78	ER (5) 0.55	SS (5) 0.56	–	4.138	0.90	0.88	0.073
Allison et al. [35]	AG (13) 0.80	DI (13) 0.74	–	–	2.459	0.91	0.90	0.050
Guan et al. [24]	EM (15) 0.86	–	–	–	4.690	0.94	0.93	0.079
Zhao et al. [17, 22, 25, 26]	EM (15) 0.86	–	–	–	4.036	0.95	0.95	0.072
This study	CE (10) 0.818	SC (8) 0.793	EE (6) 0.714	SS (5) 0.746	2.51	0.919	0.909	0.043

*CFI* comparative fit index, *TLI* Tucker–Lewis Index, *RMSEA* root mean square error of approximation, *EQ* empathy quotient, *EM* empathy, *CE* cognitive empathy, *ER* emotional reactivity, *SS* social skills, *AG* agreement, *DI* disagreement, *SC* self-consciousness, *EE* emotional empathy

In Table 5, seven structural models have been reported for the EQ, and the model description and CFA results for each model are provided. Cronbach’s *α* values for the scores on the EQ − 40 and EQ − 15 were both 0.86. The Cronbach’s *α* of every subscale of this study ranged between 0.714 and 0.818. Cronbach’s *α* values for the scores on the other EQ models are provided in Table 5. The final modified model of this study showed a good fit to the data (see Table 5).

In this modified study, the approximate values of six other structural models were gotten in a rounded way with data citations from “validation of the empathy quotient in Mainland China” [22].

## Discussion

A Chinese version of the EQ (29 items) was validated in this study with the samples of Uyghur and Hui Minority College Students in Mainland China. This study, in line with three other studies which based on Chinese populations [23–25], provides evidence to support the notion that the cognitive and emotional empathy may coexist, rather than be clearly differentiated, which was originally put forward by Baron-Cohen and Wheelwright [5].

Compared to other validations worldwide, this study also showed similarities and statistically significant gender differences in findings. Through a *T* test with independent samples, this research showed that the median scores for the total EQ as well as “self-consciousness,” “social skills,” and “emotional empathy” subscales were significantly higher in female college students compared with male college students (*P* < 0.001); however, there was no distinct difference in the scores of cognitive empathy between male and female participants. It is consistent with the findings in the majority of studies [2, 18, 26], which proved the findings of the emotion study: men are more likely to suppress their emotions while women are more inclined to express them [18]. However, it is not only completely inconsistent with Bailey’s (1996) findings and Guan’s (2012) findings [24], in which “no statistically significant gender differences were found”, but also absolutely inconsistent with Preti and Vellante’s (2011) findings, “cognitive empathy factor scores were consistently higher among females than males; there were no differences by gender on the social skills, or the emotional reactivity factor” [27]. Meanwhile, it is also different with Baron-Cohen and Wheelwright’s [5] findings, in which “sex differences (female superiority) were also found on both cognitive empathy and emotional reactivity but not on the ‘social skills’”. It might be that the participants were college students from either Hui or Uyghur nationality which were Chinese ethnic minorities in a cross-culture environment.

Dutch cultural anthropologist Hofstede described the multicultural conflicts as having four stages: curiosity, cultural disturbance, acculturation, and stabilizing [28]. The less time one spends in the stage of cultural disturbance and acculturation, the faster he or she will adapt into the new cultural environment. But the truth is that so many minority college students in Chinese Han region failed to convert this conflict due to their long time span in the stages of cultural disturbance and acculturation. Researches showed that, confronted with the dual-cultural environment, namely Han culture and their own national culture, minority college students in Chinese Han region have a sense of cultural alienation because of the friction between their mother culture and the mainstream culture of the Han nationality [29]. They have to accept the influence of Han culture on the one hand and inherit the culture of their own nationality on the other. This adapting process inevitably incurs conflicts between their native cultural position and the extraneous ones. Therefore, their scores of compulsion, depression and paranoid ideation of Xinjiang ethnic minority students in colleges of Han region were significantly higher than those of other students [30]. As a result, negative emotions such as inferiority, autism, and anxiety show up and their academic performance starts to decline, which inevitably endangers their physical and mental health. Obviously, the cultivation of empathy skills can help to shorten the time span of the cultural disturbance and acculturation stages and finally improve the ability for cross-cultural communication.

By this means, their negative emotions can be decreased progressively and their positive emotions can increase correspondingly which help individuals to become adapted to the main cultural environment in a short time. The EQ scale has first been implemented in the population of minority college students, and the cross validation for the EQ scale has been made by exploratory factor analysis and confirmatory factor analysis. Firstly, according to the exploratory factor analysis and taking into account the original structure of the EQ scale, we deleted some items, finally obtaining 29 items and four factors which have the same nomenclature as the original EQ scale. We named the four factors as cognitive empathy, self-awareness, emotional empathy, and social skills. Secondly, confirmatory factor analysis was used to verify the structural model and the results show that the model fitting is preferable. All the indicators of reliability and validity analysis showed that this version of EQ scale had a good validation.

In this study, we ranked the contribution ability of four factors in order from high to low: cognitive empathy, self-awareness, emotional empathy, and social skills. The cumulative percent of the four factors was 60.713%. This result was different from Lawrence and Baron-Cohen’s study of a British population. In their study, three factors were obtained with the contribution ability ranked in the following order (high to low): cognitive empathy, emotional empathy, and social skills. By comparison, the cumulative percent of our four factors improved upon that found in the study by Lawrence and his colleagues on the British population by 19.313%, suggesting that the EQ scale in the Chinese version is quite fit for measuring empathy in the population of Chinese Uyghur and Hui Nationality College students. Further analysis showed that in both Western and Eastern culture, cognition plays a vital role in the process of the dynamically social and psychological phenomenon of empathy. When individuals confront one or more definite emotional situations, empathy occurs according to the following steps: firstly, the emotions and feelings were shared; then, on the premise of recognizing the difference between oneself and others, cognitive assessments on the whole situation were made; consequently, the response to the emotions and feelings with appropriate actions came into being [31].

In this study, we detached a new factor, self-awareness, which plays a vital role in the empathy skills of minority college students. Compared with Han students in mainland Chinese cities, minority students’ way of thought and action on the value of orientation normally stem from their inherent cultural habits and customs, while the former have more opportunities to be well informed and to enrich social communication. The manners of thought and action of minority college students represent an unconscious status of “always being right” while never thinking “must always be right?” Consequently, this cultural and psychological structure formed the distinct psychological characteristics featured by self-centeredness, strong independence, self-respect, and sensitivity.

In addition, gender difference was scored in this revised Chinese version of the EQ scale. The results showed that there was no distinct difference in the scores of cognitive empathy between male and female participants, but the total score and scores of self-awareness, social skills, and emotional empathy were higher for females than for males. The difference of the EQ index for different genders in our study is consistent with the research results of Lawrence and Baron-Cohen [18], which proved the findings of the emotion study: men are more likely to suppress their emotions while women are more inclined to express them [32].

There are some limitations in our study. Firstly, we used the convenience sample to recruit the college students from only two universities as our research targets, so there was a sample selection bias in this study. Thus, our results may not be representative of the wider minority College Students in China. Additionally, this study used classical test theory to assess the psychometric properties of the EQ. Modern psychometric theory such as Rasch analysis was not used.

## Conclusion

In summary,this study developed a complete Chinese version of the EQ scale and found that it had a good reliability and validity among Chinese Uyghur and Hui nationality college students in the Han Chinese region. Also, the related research findings with this Chinese version EQ scale could be the reference of offering suggestions for various related educators and policy-makers to help a large quantity of minority students to improve their empathy ability and to adapt to a new learning environment much easier. A much larger number of samples will be collected, various analyzing methods such as Rasch analysis have to be used in future to verify the EQ scale structure, and an examination of factors that influence the EQ skills will be made.

## Abbreviations

EQ: empathy quotient scale
EFA: exploratory factor analysis
CFA: confirmatory factor analysis
QMEE: Questionnaire Measure of Emotional Empathy
IRI: Interpersonal Reactivity Index
PANAS: positive and negative affect scale
SSRS: social support review survey
SCSQ: simplified coping style questionnaire
CVI: content validity index
GFI: goodness of fit index
AGFI: adjusted goodness of fit index
IFI: incremental fit index value
CFI: comparative fit index
TLI: Tucker–Lewis Index
RMSEA: root mean square error of approximation
